# Diagnostic Performance of Diffusion-Weighted Imaging for Colorectal Cancer Detection: An Updated Systematic Review and Meta-Analysis

**DOI:** 10.3389/fonc.2022.656095

**Published:** 2022-06-23

**Authors:** Yunfei Xiao, Juan Li, Jiamei Zhong, Dequan Chen, Jianbo Shi, Hongrui Jin

**Affiliations:** ^1^Department of Magnetic Resonance, The First Affiliated Hospital of Zhengzhou University, Zhengzhou, China; ^2^Department of Radiology, People’s Hospital of Chongqing Hechuan, Chongqing, China; ^3^Department of Radiology, The Seventh People’s Hospital of Chongqing, Chongqing, China

**Keywords:** colorectal cancer, diagnostic performance, DWI, MRI, sensitivity, meta-analysis

## Abstract

**Background:**

Magnetic resonance imaging (MRI), which uses strong magnetic fields and radio waves (radiofrequency energy) to make images, is one of the best imaging methods for soft tissues and can clearly display unique anatomical structures. Diffusion-weighted imaging (DWI) has been developed for identifying various malignant tumors.

**Aim:**

To investigate the diagnostic value of DWI-MRI quantitative analysis in colorectal cancer detection.

**Methods:**

The PubMed, Cochrane Library, and Embase databases were searched from inception to May 29, 2020. Studies published in English that used DWI-MRI for diagnosing colorectal cancer were included. Case reports, letters, reviews, and studies conducted in non-humans or *in-vitro* experiments were excluded. The pooled diagnostic odds ratio (DOR) and hierarchical summary receiver operating characteristic (HSROC) curves were computed for DWI, and the area under the curve (AUC) and associated standard error (SE) and 95% confidence intervals (CIs) were also used.

**Results:**

In total, 15 studies with 1,655 participants were finally included in this meta-analysis. There were four prospective studies and 11 retrospective studies. Eight studies focused on rectal cancer, six on colorectal cancer, and one on colonic cancer. The performance of DWI-MRI for diagnosing colorectal cancer was accurate, with pooled sensitivity, specificity, positive likelihood ratio, and negative likelihood ratio of 0.88 (95% CI = 0.85–0.91), 0.92 (95% CI = 0.91–0.94), 30.36 (95% CI = 11.05–83.43), and 0.44 (95% CI = 0.30–0.64), respectively. The DOR and HSROC curves were 121 (95% CI = 56–261) and 0.92 (*λ*: 4.79), respectively.

**Conclusion:**

DWI showed high diagnostic accuracy for colorectal cancer detection. Further studies with large sample sizes and prospective design are needed to confirm these results.

## 1 Introduction

Colorectal cancer is one of the most common types of cancer and the second leading cause of cancer-related mortality worldwide (1). The early diagnosis of colorectal cancer is crucial to prevent metastasis and reduce the fatality rate. Magnetic resonance imaging (MRI) is one of the best imaging methods for soft tissues and can clearly display unique anatomical structures. MRI can provide quantitative and qualitative information about the integrity of cell membranes and tissues. MRI can be used for initial diagnosis to determine the treatment plan for colorectal cancer (2). With the continuous development of MRI technology, the specificity and the diagnostic accuracy of traditional MRI for the detection of colorectal cancer have been largely improved (3).

Diffusion-weighted imaging (DWI) is a recently developed functional MRI imaging technique based on the movement of water molecules rather than the anatomical structure (4). Over the past decade, DWI has been widely used in the clinical setting for the early diagnosis of various cancers, such as breast cancer (4), prostatic cancer (5), and cervical cancer (6).

Several recent studies (7–10) have demonstrated that DWI provides high-contrast resolution morphological imaging, thereby significantly improving the diagnostic accuracy of colorectal cancer by MRI. In 2015, a meta-analysis (11) reported that DWI could be a highly accurate diagnostic method for the identification of colorectal cancer. However, previous meta-analyses included studies with small sample sizes published before May 2015, and stratified analyses could not be performed to draw a more detailed conclusion. Several recent studies with a larger sample size have provided new perspectives on this topic, and the reported DWI diagnostic performance was inconsistent with previous findings (12, 13). Given the recent evidence on the high accuracy of DWI in the preoperative diagnosis of colorectal cancer and the initial detection of colorectal neoplasms (14), an updated systematic review and meta-analysis was needed, which might provide specific suggestions for the early clinical diagnosis of colorectal cancer. Therefore, we conducted this meta-analysis to investigate the diagnostic performance of DWI-MRI for colorectal cancer detection.

## 2 Materials and Methods

The current study was performed according to the guidelines of the 2009 Preferred Reporting Items for Systematic Reviews and Meta-Analysis (PRISMA) statement (15).

### 2.1 Literature Search

The following individual and joint keywords “diffusion-weighted imaging,” “DWI,” “colorectal OR colon OR rectal OR CRC,” and “tumor OR cancer OR carcinoma OR neoplasm” were used for searching studies published in English in the PubMed, Cochrane Library, and Embase databases from inception to May 29, 2020. To identify additional relevant studies, the bibliographies of all relevant studies and reviews were manually searched, and Google Scholar was also searched for additional publications that cited relevant studies.

### 2.2 Eligibility Criteria

Studies were eligible for inclusion in this meta-analysis if they fulfilled the following criteria: 1) cases with a histologically confirmed diagnosis of colorectal cancer; 2) colorectal cancer detection or staging by DWI-MRI; 3) studies providing sufficient raw data to construct a 2 × 2 contingency table to perform MRI sequences; 4) studies published in English; and 5) for studies conducted using the same study participants, the most recent publication or the one with more detailed information was included.

Case reports, letters, reviews, studies conducted in non-humans or *in-vitro* experiments, studies not published in English, and studies for which data were not available were excluded from the analysis.

### 2.3 Data Extraction

All eligible studies were independently assessed by two reviewers. Any disagreement between them on study inclusion was resolved after consultation with a third reviewer. The data from the included studies were extracted using a standardized form, and consensus was reached on all items by the two reviewers. The following data were extracted: study characteristics (e.g., authors, year of publication, country, sample size, and study design), patient characteristics (e.g., mean age and female percentage), and imaging characteristics including field strength, type of coil, MR sequences and features, and the true-negative (TN), false-negative (FN), true-positive (TP), and false-positive (FP) values.

### 2.4 Methods of DWI-MRI Included

There are different methods in the field of DWI. In the current study, we assessed the diagnostic performance of DWI based on apparent diffusion coefficient (ADC) measurement, which relies on the ADC changing with the diffusion time as measurements move from restricted to free diffusion regimes.

### 2.5 Assessment of the Quality of Studies

The study quality was independently assessed and cross-checked by the two reviewers as per the Quality Assessment of Diagnostic Accuracy Studies 2 (QUADAS-2) tool (16). The QUADAS-2 comprises four domains: 1) patient selection, 2) index test, 3) reference standard, and 4) flow and timing.

### 2.6 Statistical Analysis

The sensitivity and specificity of DWI were calculated based on the TN, FN, TP, and FP values. The data from each study were pooled by using a random-effects model and expressed with the corresponding 95% confidence intervals (CI). The pooled diagnostic odds ratio (DOR) and hierarchical summary receiver operating characteristic (HSROC) curves were computed for DWI, and the area under the curve (AUC) and associated standard error (SE) and 95% CIs were also calculated. The DerSimonian and Laird random-effects method was employed to determine the AUC of the SROC curves due to the presence of heterogeneity. The 95% CIs of the pooled metrics were compared to assess the relative diagnostic performance of the technique.

The *I*^2^ statistics were used to assess the consistency of the effect sizes, which subsequently indicated the percentage of variability in effect estimates because of true interstudy variance rather than intrastudy variance. Heterogeneity was defined as low, moderate, and high by the *I*^2^ values of 25%, 5%, and 75%, respectively (17). Publication bias was assessed by Begg’s rank correlation (18) and Egger’s weighted regression methods (19) (*p *< 0.05 indicated significant publication bias). Stata 15.0 (Stata Corporation, College Station, TX, USA) was used for the statistical analyses. The DOR and HSROCs were assessed with “metandi” modules in Stata software. A *p-*value <0.05 indicated a significant difference for all the analyses.

## 3 Results

### 3.1 Study Selection

**Figure 1:**
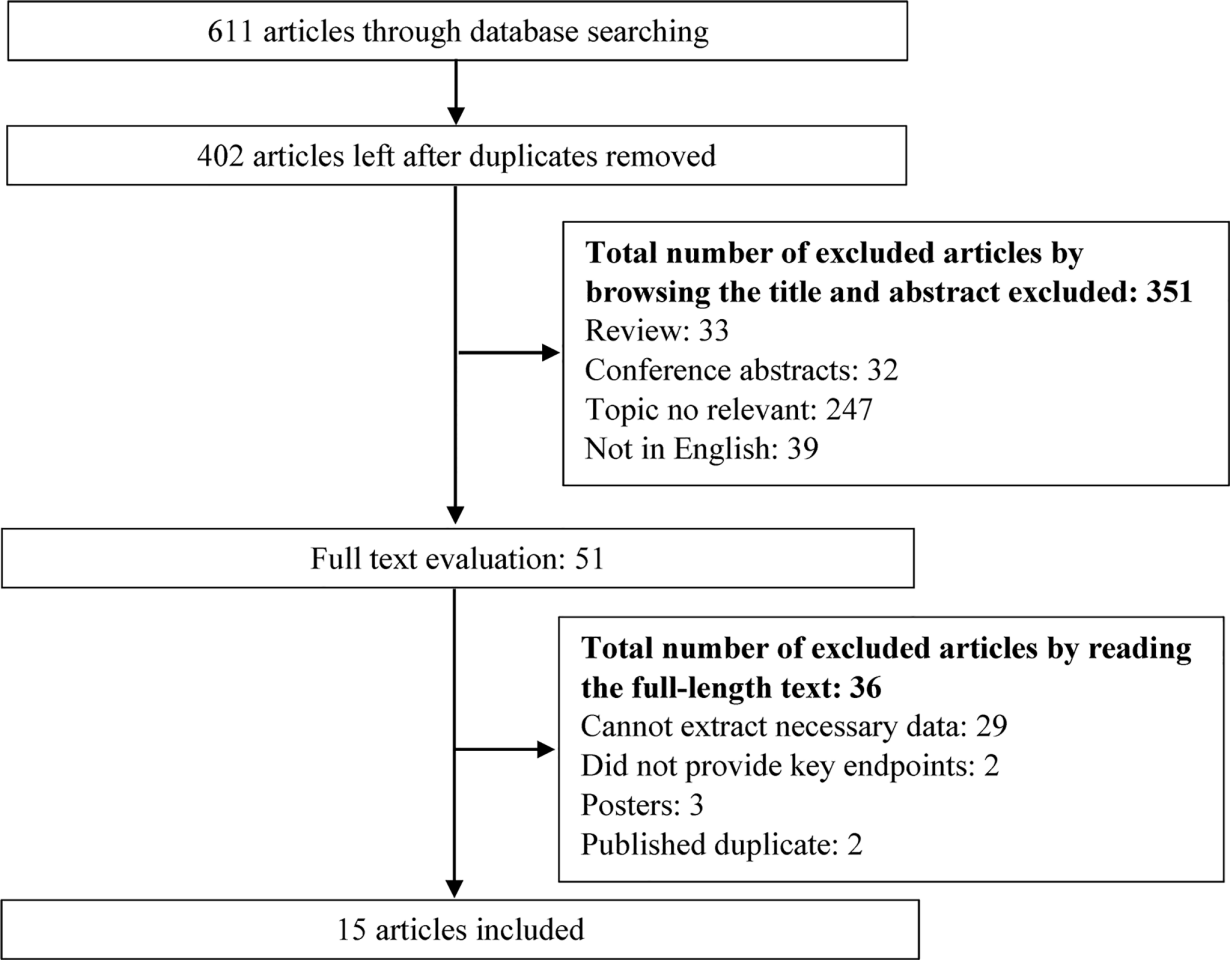
Flowchart of the study selection process.

The search strategy yielded 611 potentially relevant studies, of which 209 were excluded due to overlapping data. Of the remaining 402 studies, 351 studies were excluded by browsing the titles or abstracts. Finally, 15 articles (7–10, 14, 20–28) were included for data extraction and meta-analysis after reading the full texts. The flowchart of the study selection process is shown in **Figure 1**.

### 3.2 Study Characteristics

In total, 15 studies with 1,655 participants were finally included in this meta-analysis. The characteristics of the included studies and participants are listed in **Tables 1**, **2**. The studies were published between 2006 and 2020. Four studies were conducted in Turkey (8, 24–26); two each in China (7, 29), Japan (14, 22), France (10, 23), and Netherlands (9, 13); and one each in Italy (20), Germany (27) and India (21). Four studies were prospective, and the diagnoses of the all cases were confirmed by histopathological examination. Eight studies focused on rectal cancer, six on colorectal cancer, and one on colonic cancer. Majority of the studies involved phased-array body coil and all studies used 1.5-T MRI. Two studies (24, 29) provided the results from one radiologist and the other studies involved two or three independent radiologists.

**Table 1:** Characteristics of the studies.

Studies included	Country	Cases		Control		Location of cancer	MRI	Case diagnosis method	Control	Study design
		Age^a^	M/F	Age^a^	M/F					
(14)	Japan	64/31–81	11/4	65/51–81	14/4	CR	Siemens AG	Histopathology	Other cancer cases	Prospective
(22)	Japan	59/33–69	18/15	NA	NA	R	Siemens Healthcare	Histopathology	Colonoscopy-negative patients	Retrospective
(7)	China	61/21–86	23/22	NA	NA	R	Siemens AG	Histopathology	Colonoscopy-negative patients	Prospective
(8)	Turkey	57/NA	NA	45/NA	NA	CR	Siemens Healthcare	Histopathology	Colonoscopy-negative patients	Retrospective
(23)	France	69/43–84	14/17	65/30–81	14/17	CR	Siemens Magnetom	Histopathology	Without recurrence cases	Prospective
(9)	Netherlands	68/35–87	13/6	64/22–81	13/10	CR	GE Healthcare	Histopathology	Without recurrence cases	Retrospective
(24)	Turkey	57/31–77	32/0	NA	NA	R	Siemens Healthcare	Histopathology	Healthy subjects	Retrospective
(25)	Turkey	63/30–81	12/5	61/46–69	10/3	CR	Siemens Magnetom	Histopathology	Without recurrence cases	Prospective
(26)	Turkey	NA	NA	NA	NA	R	Siemens Magnetom	Histopathology	Benign cases	Retrospective
(10)	France	64/NA	17/10	69/NA	14/8	R	Philips Medical	Histopathology	IBD cases	Retrospective
(27)	Germany	63/29–87	57/15	NA	NA	R	Siemens Magnetom	Histopathology	Scar tissue	Retrospective
(13)	Netherlands	65/32–84	NA	NA	NA	R	Philips Healthcare	Histopathology	Without recurrence cases	Retrospective
(20)	Italy	64	12/6	63.76	20/5	R	Siemens Magnetom	Histopathology	Fibrosis	Retrospective
(29)	China	NA	NA	27–67	NA	C	Siemens Healthcare	Histopathology	Para-carcinoma tissue	Retrospective
(21)	India	47/22–70	26/11	48/19–86	138/76	CR	Philips Healthcare	Histopathology	Colonoscopy-negative patients	Retrospective

MRI, magnetic resonance imaging; M, male, F, female; CR, colorectal; R, rectal, C, colonic; IBD, inflammatory bowel disease; NA, not available.

a Mean/range, years.

**Table 2:** Characteristics of magnetic resonance imaging.

Studies included	Cases/controls	No. of readers	Coil type	Magnetic	Methods	ADC value (mm^2^/s)		*β* value	Method of analysis
						Patients	Control		
(14)	15/20	2	Body and spine matrix coil	1.5 T	HSI, ADC	1.19	1.37	0–500–1,000	Visual
(22)	31/31	2	Anterior torso phased-array coil	1.5 T	HSI, ADC	NA	NA	0–1,000	Visual
(7)	33/15	3	Phased-array body coil	1.5 T	ADC	NA	NA	0–500–1,000	Visual
(8)	45/20	2	Abdominal phased-array coil	1.5 T	ADC	1.07	1.91	0–800	Visual
(23)	42/22	3	Phased-array body coil	1.5 T	ADC	1.04	1.39	0–1,000	Non-visual
(9)	19/23	2	Phased-array body coil	1.5 T	ADC	0.97	1.37	0–500–1,000	Non-visual
(24)	23/30	1	Phased-array body coil	1.5 T	ADC	1.02	1.53	0–800	Non-visual
(25)	17/13	2	Phased-array body coil	1.5 T	HIS, ADC	1.19	2.69	0–800	Visual
(26)	26/15	2	Phased-array body coil	1.5 T	ADC	NA	NA	0–800	Visual
(10)	27/31	2	Phased-array body coil	1.5 T	HSI	NA	NA	0–500–1,000	Visual
(27)	23/49	2	Phased-array body coil	1.5 T	ADC	1.02	1.77	0–100–400–800–1,000	Non-visual
(13)	12/428	2	Phased-array body coil	1.5 T	ADC	NA	NA	0–1,000	Visual
(20)	18/25	3	Phased-array body coil	1.5 T	ADC	1.22	1.6	0–800	Non-visual
(29)	186/165	1	Phased-array body coil	1.5 T	ADC	NA	NA	0–800	Visual
(21)	37/214	2	Phased-array body coil	1.5 T	ADC	NA	NA	0–400–800	Visual

HSI, lesions were identified as malignant with the appearance of focal areas of high-signal intensity; ADC, lesions were identified as malignant with significantly lower ADC value; NA, not available.

### 3.3 Assessment of Study Quality and Risk of Bias

The two reviewers independently evaluated each included study according to the QUADAS-2 items. None of the items of the QUADAS-2 was judged as high risk. The quality assessment results are presented in **Supplementary Figure 1**.

### 3.4 Diagnostic Accuracy

**Figure 2:**
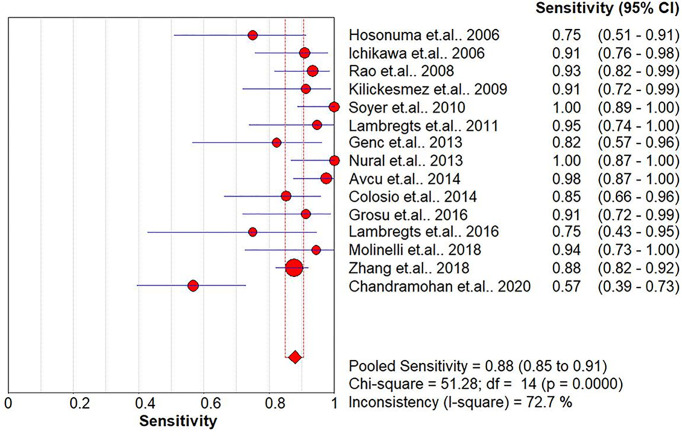
Summary of the sensitivity analysis of the included studies.

**Figure 3:**
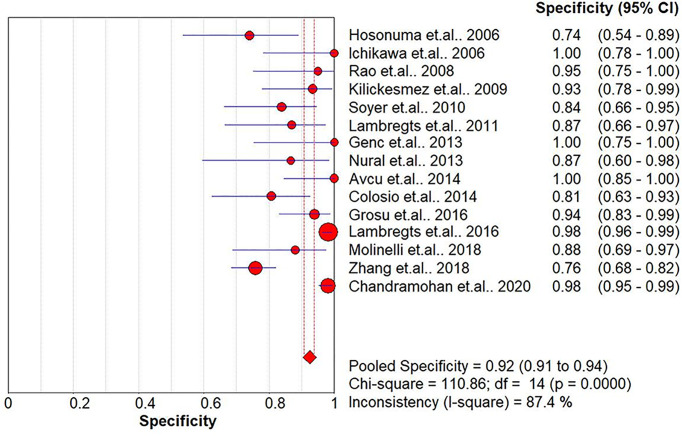
Summary of the specificity analysis of the included studies.

**Figure 4:**
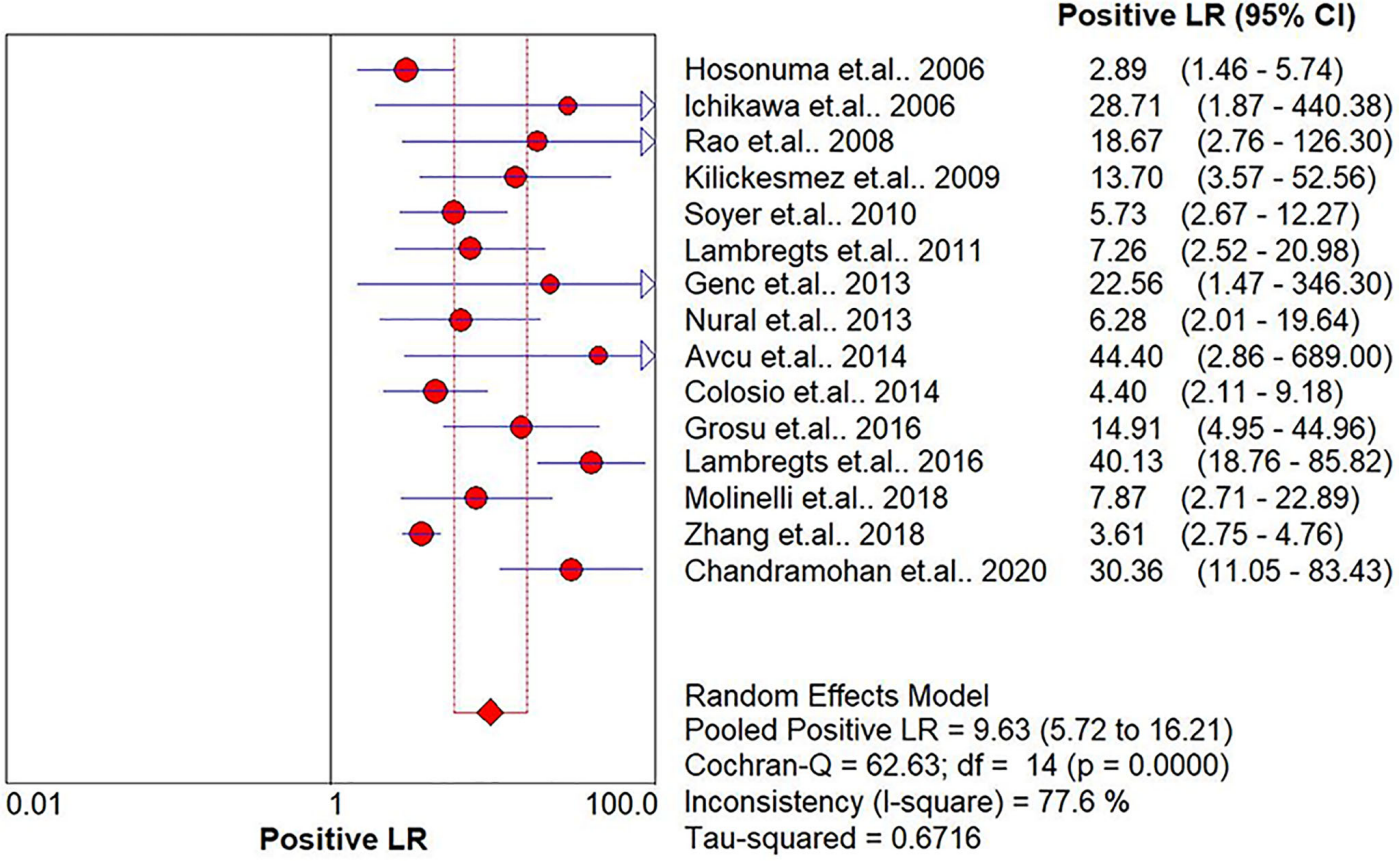
Summary of the positive likelihood ratio of the included studies.

**Figure 5:**
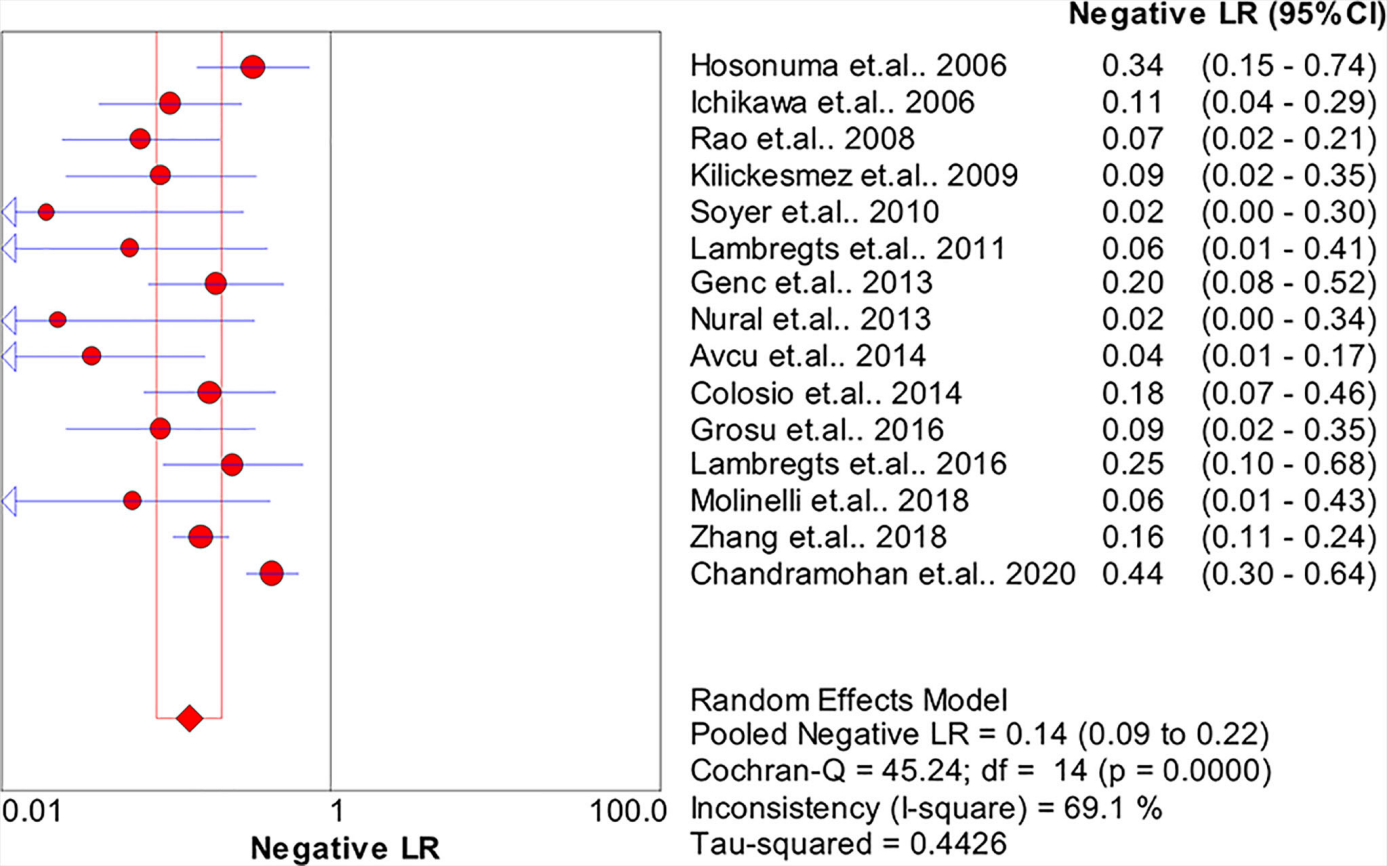
Summary of the negative likelihood ratio of the included studies.

**Figure 6:**
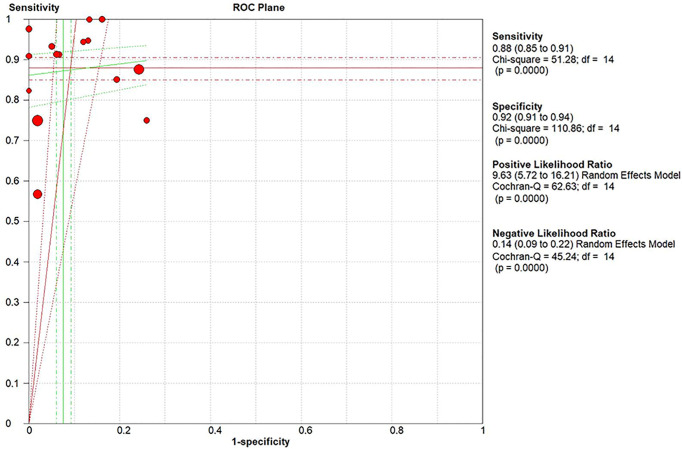
Summary of the pooled ROC plane.

**Figure 7:**
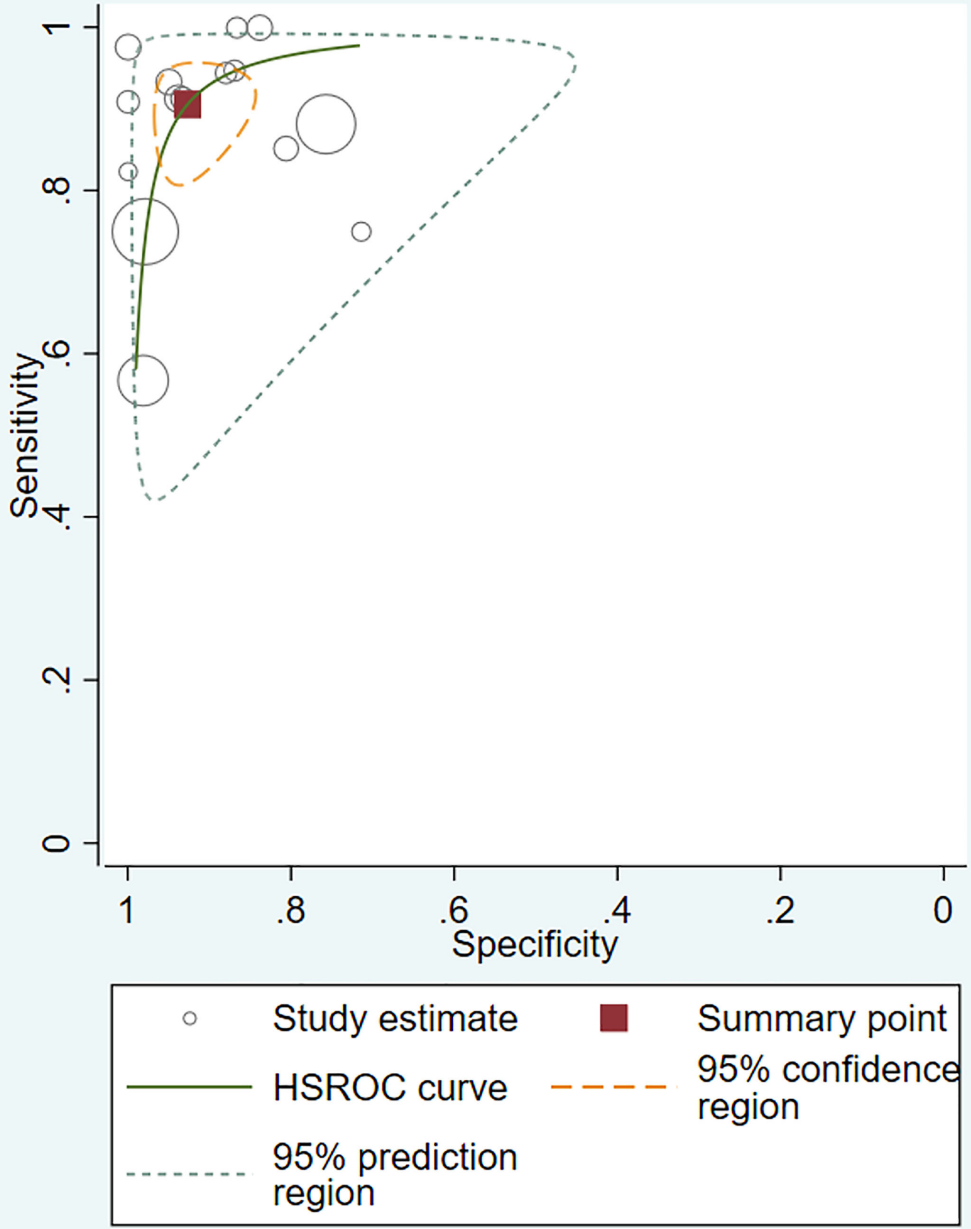
Pooled hierarchical summary receiver operating characteristic (HSROC) curves.

The pooled sensitivity, specificity, positive likelihood ratio (PLR), negative likelihood ratio (NLR), and HSROC for the diagnostic accuracy of DWI are presented in **Figures 2**–**7**, respectively. The DWI exam showed high diagnostic accuracy for colorectal cancer, with a sensitivity of 0.88 (95% CI = 0.85–0.91), a specificity of 0.92 (95% CI = 0.91–0.94), a PLR of 30.36 (95% CI = 11.05–83.43), and an NLR of 0.44 (95% CI = 0.30–0.64). The pooled DOR (121, 95% CI = 56–261) and HSROC (0.92, *λ* = 4.79) were consistent with the predicted results for sensitivity, specificity, PLR, and NLR. The forest plots suggested that heterogeneity was high, with almost all *I*^2^ values >8%.

### 3.5 Subgroup Analysis

In **Table 3**, we presented the results on subgroups to explore the effect of study design and geographical regions for sampling on pooled sensitivity, specificity, PLR, NLR, and DOR. Five studies reported data on participants from Asian countries, and four were prospective studies. The pooled results for the studies grouped by study design showed no significant change when compared to the results of all the other studies. As the studies were categorized by country, the results for studies from non-Asian countries showed slightly better pooled sensitivity, specificity, PLR, and NLR. As shown in **Table 3**, by dividing the studies into various groups, the heterogeneity for sensitivity, specificity, PLR, NLR, and DOR was decreased to a moderate level (*I*^2^* *< 75%). Moreover, we conducted a subgroup analysis with the inclusion of papers published after 2015, and the results are comparable with the results from all studies (**Supplementary Figure 2**).

**Table 3:** Subgroup analyses of the diagnostic performance of DWI.

Studies	No. of studies	Sensitivity (95% CI), *I*^2^	Specificity (95% CI), *I*^2^	PLR (95% CI), *I*^2^	NLR (95% CI), *I*^2^	Diagnostic OR (95% CI), *I*^2^
All studies	15	0.88 (0.85–0.91), 72.7%	0.92 (0.91–0.94), 87.4%	30.36 (11.05–83.43), 77.6%	0.44 (0.30–0.64), 69.1%	68.91 (21.09–225.13), 55.6%
Study design						
Retrospective	11	0.88 (0.84–0.91), 76.6%	0.93 (0.91–0.94), 89.4%	30.36 (11.05–83.43), 81.3%	0.44 (0.30–0.64), 72.7%	68.91 (21.09–225.13), 49.6%
Prospective	4	0.89 (0.87–1.00), 64.2%	0.91 (0.82–0.96), 81.3%	14.34 (1.42–144.60), 79.4%	0.15 (0.06–0.35), 63.5%	NA
Country						
Asian	5	0.84 (0.80–0.88), 82.8%	0.88 (0.85–0.91), 93.2%	8.29 (3.14–21.89), 82.5%	0.20 (0.10–0.38), 82.4%	40.54 (14.20–115.73), 65.8%
Others	4	0.93 (0.89–0.96), 50.8%	0.95 (0.93–0.97), 73.7%	10.40 (5.93–18.27), 61.3%	0.11 (0.07–0.19), 25.5%	111.80 (57.72–216.56), 0.0%

CI, confidence interval; PLR, positive likelihood ratio; NLR, negative likelihood ratio; OR, odds ratio; NA, not available.

### 3.6 Publication Bias

No potential publication bias was observed among the included trials (all *p-*values > 0.05) according to Begg’s rank correlation analysis and Egger’s weighted regression analysis. The detailed potential publication bias is presented in **Supplementary Table 1**.

## 4 Discussion

The current study systemically explored the diagnostic accuracy of DWI in detecting colorectal cancer. Four prospective and 11 retrospective studies were included in this meta-analysis. DWI showed good diagnostic ability for colorectal cancer detection, with pooled sensitivity, specificity, PLR, and NLR of 0.88 (95% CI = 0.85–0.91), 0.92 (95% CI = 0.91–0.94), 30.36 (95% CI = 11.05–83.43), and 0.44 (95% CI = 0.30–0.64), respectively, and the pooled area under the SROC curve was 0.9654. When the studies were categorized by country, the studies from non-Asian countries showed slightly higher diagnostic power.

Another meta-analysis (11) investigated DWI for colorectal cancer detection and showed a pooled sensitivity and specificity of 0.95 (95% CI = 0.90–0.97) and 0.93 (95% CI = 0.85–0.97), respectively. In the current study, five updated studies were counted, and in total, 15 studies were included. Compared with a previous meta-analysis, the five newly included studies had larger sample sizes, and finally, the number of study subjects was almost twice that of the previous one. We observed a similar diagnostic performance of DWI with the previous meta-analysis. Compared with the traditional MRI, DWI might obtain a better performance, and better diagnostic performance was also observed in other types of cancers, such as uterine cervical cancer (12), prostate cancer (30), and esophageal cancer (30). A study focused on cervical cancer (30) and the results demonstrated that the overall benefits of using DWI in 3.0-T MRI resulted in higher reader confidence, sensitivity of tissue infiltration, and tumor grading. Another meta-analysis (31) indicated that the diagnostic capacity of 1.5- and 3.0-T MRI scanners for prostatic cancer was comparable. In the current study, all patients were evaluated by 1.5-T MRI for colorectal cancer. In the future, a comparison of the diagnostic performance of 1.5- and 3.0-T MRI for colorectal cancer is needed. The recently developed functional MRI techniques provide high-contrast resolution morphological imaging, metabolic information, and direct depiction of tumor vascularity. The multiparametric MRI methods, such as DCE-MRI or combined DWI and DCE-MRI, might improve the sensitivity and specificity of detection and determination of tumor staging for colorectal cancer (32, 33).

One of the major aims of the colorectal cancer MRI test is to determine the tumor staging rather than tumor presence. This may be the reason that only a few studies have investigated DWI for the initial detection of colorectal cancer. In clinical settings, it is necessary to locate the tumor by MRI, and DWI may be helpful in the diagnosis of some particularly difficult cases, such as for smaller tumors or when the tumors are obscured by feces. Up to now, few studies have focused on the role of DWI in the staging of colorectal cancer. One study conducted by Lu et al. (34) reported that DWI was useful to evaluate the T stage of rectal cancer. Lymph node staging remains the most challenging task in clinical practice. Therefore, the implementation of DWI for lymph node staging is appealing. Since the value of DWI characterization for lymph nodes is still undefined (35), more applied clinical research on DWI is warranted in this area in the future.

The strengths of our study are the systemic search of potential studies in several popular databases and the high quality of the included studies. However, we need to consider the limitations of the present meta-analysis when interpreting the results. First, the studies included in the current meta-analysis were limited and the majority of them had small sample sizes. The small number of participants might reduce the credibility and stability of the results. Moreover, the small sample sizes also limited further subgroup analyses or sensitivity analyses, e.g., only a few studies on colon cancer were available, and MRI with DWI was reported to have a better performance in detecting more colonic cancers than CT (36). Second, some of the studies did not match the participants by age or gender. Therefore, the mean age and sex ratios of the participants among the included studies varied largely, which might also cause heterogeneity and reduce the stability of the results. Third, the included studies used varied controls, which might be one of the main reasons for the high heterogeneity. For the subgroup analysis, heterogeneity was decreased to a moderate level. Fourth, the technical factors for DWI might also affect the results of perfusion imaging. However, due to the limited number of the included studies, we could not conduct further analysis of the technical factors, such as the rate of contrast media injection and patients’ hemodynamic status. Fifth, due to insufficient data information, we could not report more results beyond tumor identification, such as detection of tumor margins. Sixth, although DWI might show diagnostic accuracy for colorectal cancer, in actual clinical practice, it might be affected by many factors, such as the varying experiences of the radiologists and the variations in image quality. This might be one obstacle in the implementation of DWI for the detection of colorectal cancer. Seventh, the effects of screening were likely to be overestimated results from publication bias, even though no publication bias was seen by both Begg’s and Egger’s tests. Finally, potential language bias might exist as our literature search included articles published in English only.

## 5 Conclusion

In summary, using DWI for the diagnosis of colorectal cancer might yield accurate AUC, sensitivity, and specificity. In the future, more studies addressing this topic with large sample sizes are warranted to confirm these results. The diagnostic performance of DWI for the detection of other cancers needs to be further evaluated and compared.

## Data Availability Statement

The original contributions presented in the study are included in the article/**Supplementary Material**. Further inquiries can be directed to the corresponding author.

## Author Contributions

Conception and design of the study: YX and HJ. Administrative support: YX and HJ. Provision of the study materials or patients: YX, JL, JZ, DC, JS, and HJ. Collection and assembly of data: YX, JL, JZ, DC, JS, and HJ. Data analysis and interpretation: YX and HJ. Writing and final approval of the manuscript: YX, JL, JZ, DC, JS, and HJ.

## Conflict of Interest

The authors declare that the research was conducted in the absence of any commercial or financial relationships that could be construed as a potential conflict of interest.

## Publisher’s Note

All claims expressed in this article are solely those of the authors and do not necessarily represent those of their affiliated organizations, or those of the publisher, the editors and the reviewers. Any product that may be evaluated in this article, or claim that may be made by its manufacturer, is not guaranteed or endorsed by the publisher.

## Supplementary Material

Supplementary Figure 1 Risk of bias assessment of included studies.

Supplementary Figure 2 **(A)** Summary sensitivity analysis of the included studies published after 2015. **(B)** Summary specificity analysis of the included studies published after 2015. **(C)** Summary of positive likelihood ratio of the included studies published after 2015. d. Summary of negative likelihood ratio of the included studies published after 2015.
