# Supplementary figures and images for: Diagnostic Performance of Diffusion-Weighted Imaging for Colorectal Cancer Detection: An Updated Systematic Review and Meta-Analysis
Source: Front Oncol. 2022 Jun 23;12:656095. doi: 10.3389/fonc.2022.656095 (PMC9260027; doi:10.3389/fonc.2022.656095)

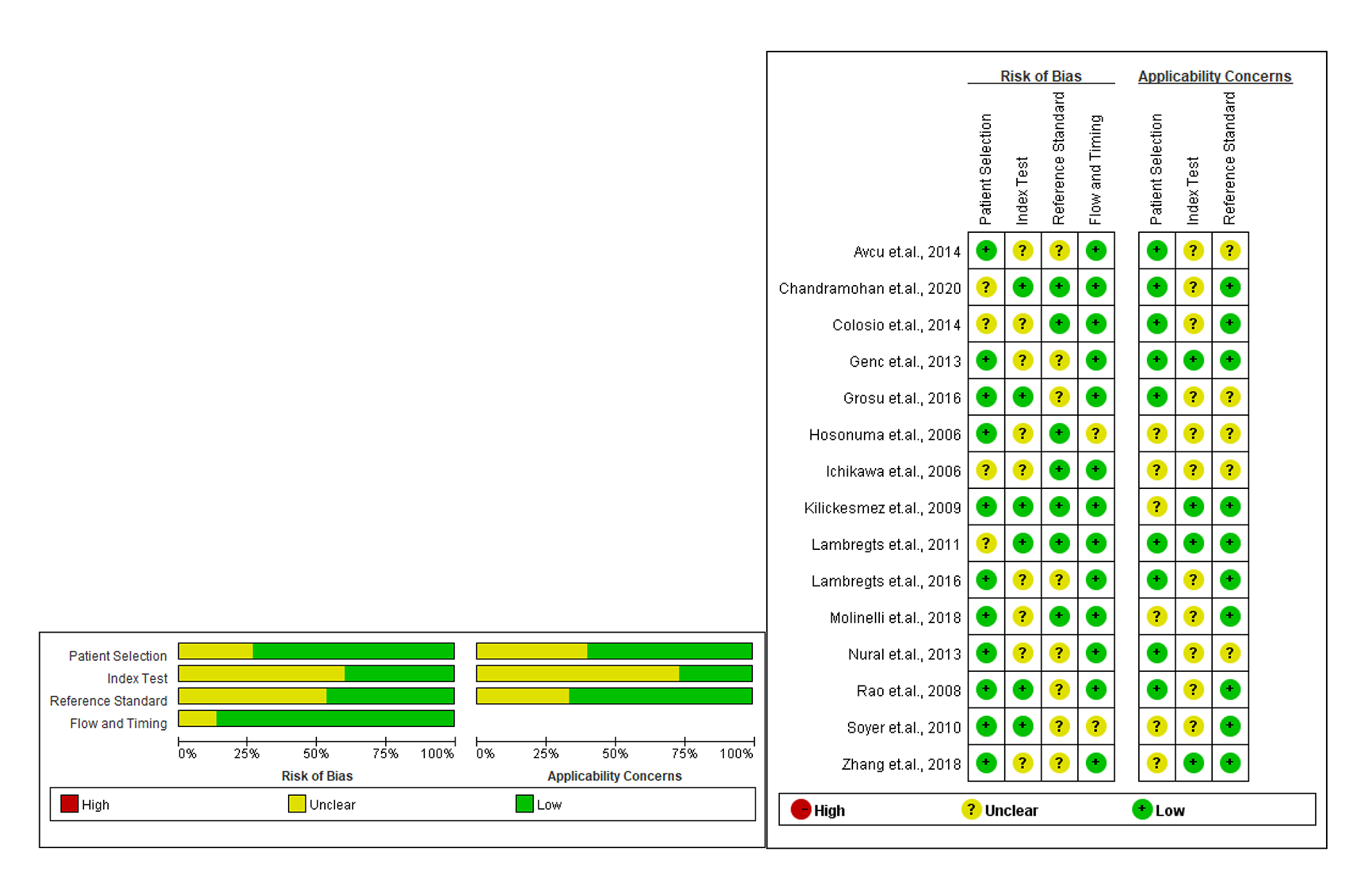

Supplement: Supplementary Figure 1 — Risk of bias assessment of included studies. [file Image_1.tiff]

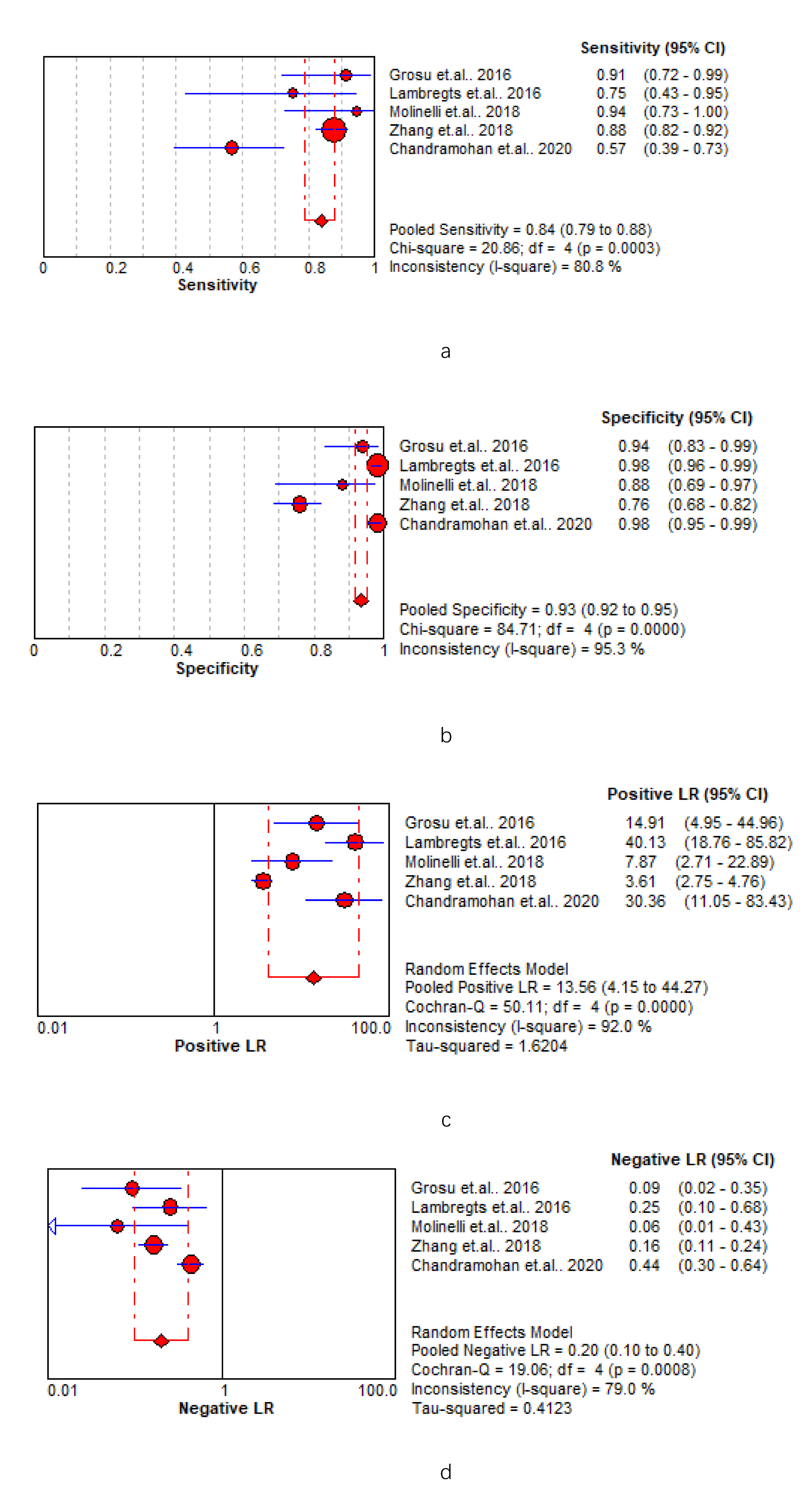

Supplement: Supplementary Figure 2 — (A) Summary sensitivity analysis of the included studies published after 2015. (B) Summary specificity analysis of the included studies published after 2015. (C) Summary of positive likelihood ratio of the included studies published after 2015. d. Summary of negative likelihood ratio of the included studies published after 2015. [file Image_2.jpeg]
