# Supplementary material for: Diagnostic Performance of Diffusion-Weighted Imaging for Colorectal Cancer Detection: An Updated Systematic Review and Meta-Analysis
Source: Front Oncol. 2022 Jun 23;12:656095. doi: 10.3389/fonc.2022.656095 (PMC9260027; doi:10.3389/fonc.2022.656095)
Supplement: Supplementary file 3 [file Table_1.docx]

Table S1. Publication bias of summarized outcomes

| **Outcomes** | **Begg (*P* value)** | **Egger (*P* value)** |
| --- | --- | --- |
| Summarized sensitivity | 0.61 | 0.44 |
| Summarized specificity | 0.61 | 0.36 |
| Summarized positive likelihood ratio | 0.56 | 0.32 |
| Summarized negative likelihood ratio | 0.75 | 0.41 |
| Summarized diagnostic odds ratios | 0.45 | 0.40 |
| Summarized pooled area under the SROC | 0.81 | 0.51 |

SROC: summary receiver operating characteristic curve
